# Suppressed Cellular Senescence Mediated by T-box3 in Aged Gastric Epithelial Cells may Contribute to Aging-related Carcinogenesis

**DOI:** 10.1158/2767-9764.CRC-22-0084

**Published:** 2022-08-08

**Authors:** Akio Takeuchi, Naoki Asano, Akira Imatani, Masashi Saito, Xiaoyi Jin, Masahiro Saito, Takeshi Kanno, Waku Hatta, Kaname Uno, Tomoyuki Koike, Atsushi Masamune

**Affiliations:** Division of Gastroenterology, Tohoku University Graduate School of Medicine, Miyagi, Japan.

## Abstract

Aging is a risk factor for cancers in various organs. Recent advances in the organoid culturing system have made it viable to investigate the influence of aging utilizing these mini organs. In this study, we aimed to examine the implications of aging for gastric carcinogenesis. Gastric organoids established from aged mice grew larger, proliferated vigorously, and survived longer than that from young mice. Because Wnt/β-catenin signaling was intensified in the aged organoids and because removal of Wnt-related factors diminished their proliferation, we investigated for Wnt target gene that contributed to enhanced proliferation and discovered that the aged organoids expressed the transcription factor T-box3 (Tbx3), which has been reported to suppress cellular senescence. Indeed, cellular senescence was suppressed in the aged organoids, and this resulted from enhanced G_2_–M transition. As for the mechanism involved in the intensified Wnt/β-catenin signaling, we identified that Dickkopf3 (Dkk3) expression was reduced in the aged organoids due to methylation of the *Dkk3* gene. Finally, the expression of TBX3 was enhanced in human atrophic gastritis and even more enhanced in human gastric cancers. In addition, its expression correlated positively with patients’ age. These results indicated that the emergence of antisenescent property in aged gastric organoids due to enhanced Tbx3 expression led to accelerated cellular proliferation and organoid formation. Because the enhanced Tbx3 expression seen in aged gastric organoids was also observed in human gastric cancer tissues, this Dkk3-Wnt-Tbx3 pathway may be involved in aging-related gastric carcinogenesis.

**Significance:**

This work provides an insight into the mechanism involved in aging-related gastric carcinogenesis through studies utilizing organoids established from young and aged murine stomachs.

## Introduction

Aging is regarded as one of the main causes of cancers in various organs, and it is well established that morbidity of cancers, including gastric cancers, increases with age (1). The progression of atrophic gastritis and intestinal metaplasia with age in *Helicobacter pylori* (*H. pylori*) infected stomach is considered as one of the causes of gastric cancers (2, 3). We have previously reported that chronic *H. pylori* infection in mice induced intestinal metaplasia, a precancerous lesion, in their stomach via induction of caudal type homeobox 2 (4). In this previous study, we discovered that innate immunity-related molecule was playing a pivotal role in the induction of precancerous changes in the stomach. This previous study gave rise to our next question, which is the molecular mechanism in which aging causes gastric carcinogenesis.

López-Otín and colleagues reported the hallmarks of aging such as genomic instability, telomere attrition, epigenetic alterations, mitochondrial dysfunction, and cellular senescence (5). Among these, cellular senescence is an irreversible cell-cycle arrest during G_1_–S or G_2_–M cell-cycle phase (6). It functions as a defense against carcinogenesis by removing senescent cells resulting from cellular injuries such as oxidative stress, DNA injury, and activation of oncogenes, as well as telomere shortening (7).

Lately, stem cell aging, the age-related deterioration of stem cell function, has become considered as one of the causes of aging (8), and morbid stem cell aging is considered to contribute to carcinogenesis (9). Although Wnt/β-catenin signaling pathway plays a crucial role in maintaining and regulating stem cells (10), the same pathway is also involved in the maintenance and the growth of cancer cells (11), which suggests the involvement of this pathway in stem cell–derived carcinogenesis, as is seen with morbid aging stem cells.

Recent advances in the manipulation of Wnt/β-catenin signaling have enabled scientists to establish organoids from adult tissue stem cells in a three-dimensional culture (12). Organoids consist of organ-specific stem cells and stem cell–derived cells, and they possess the organ-specific function and structure (13).

Studies with gastric organoids have made advances in clarifying the differentiation and carcinogenesis of gastric cancers (14, 15); however, it is still unclear how aging affects gastric carcinogenesis. In this study, we aimed to elucidate the mechanism involved in aging-related gastric carcinogenesis by utilizing organoids established from young and aged murine stomachs.

## Materials and Methods

### Cell Line and Animals

Human gastric cancer cell line AGS and human embryonic kidney cell line HEK were purchased from ATCC where cell lines had been authenticated by short tandem repeat profiling. Cell lines were maintained according to the distributor's protocols and were carefully checked for morphologic consistency by microscope. Cultures of cell lines were checked for *Mycoplasma* contamination by Cycleave PCR *Mycoplasma* Detection Kit (TaKaRa Bio). C57BL/6 mice were purchased from CLEA Japan and reared in the animal facility in Tohoku University Graduate School of Medicine (Miyagi, Japan). Mice were handled according to the Regulations for Animal Experiments and Related Activities at Tohoku University (Miyagi, Japan). According to a previous report from Nalapareddy and colleagues (16), mice of 2 to 4 months of age were defined as young mice, whereas that of more than 18 months of age were defined as aged mice.

### Antibodies

Antibodies used in this study are listed in Table 1.

**TABLE 1:** Antibodies used in this study

Immunogen	Source	Supplier	Dilution
p16^INK4A^	Rabbit	Cell Signaling Technology (#92803)	Immunofluorescence 1:800
p19^ARF^	Rat	Invitrogen (#MA1-16665)	Immunofluorescence 1:500
p21^WAF1/CIP1^	Rabbit	Cell Signaling Technology (#2947)	Immunofluorescence 1:400
p53	Mouse	Cell Signaling Technology (#2524)	Immunofluorescence 1:2,000
Tbx3	Rabbit	Abcam (#ab99302)	Immunofluorescence 1:200 Western blot analysis 1:1,000 IHC 1:250
Dkk3	Rabbit	Abcam (#ab186409)	IHC 1:200
Ki-67	Rabbit	Nichirei (#418071)	Immunofluorescence prediluted
Ki-67	Mouse	Dako Cytomation (#M7240)	IHC 1:200
CD324 (E-Cadherin) (Alexa Fluor 488 Conjugate)	Rat	Invitrogen (#53-3249-82)	Immunofluorescence 1:200
Rabbit IgG (H+L), F(ab')_2_ Fragment (Alexa Fluor 555 Conjugate)	Goat	Cell Signaling Technology (#4413)	Immunofluorescence 1:100
Mouse IgG (H+L), F(ab')_2_ Fragment (Alexa Fluor 555 Conjugate)	Goat	Cell Signaling Technology (#4409)	Immunofluorescence 1:100
Rat IgG (H+L), F(ab')_2_ Fragment (Alexa Fluor 555 Conjugate)	Goat	Cell Signaling Technology (#4417)	Immunofluorescence 1:100
Actin	Rabbit	Santa Cruz Biotechnology (#sc-1616-R)	Western blot analysis 1:5,000
Rabbit IgG (HRP-linked)	Goat	Cell Signaling Technology (#7074)	Western blot analysis 1:2,000
Mouse IgG (MAX-PO-linked)	Goat	Nichirei (#424131)	IHC prediluted
Rabbit IgG (MAX-PO-linked)	Goat	Nichirei (#424141)	IHC prediluted

### Gastric Organoid Culture

Gastric organoids were established according to a previous report from Nanki and colleagues (14). Briefly, stomachs removed from mice were minced with surgical scissors and processed by Liberase TH (Roche Diagnostics). The processed cells were suspended in Matrigel (Corning), plated onto 48-well multiple-well plates, and cultured in WRC+ medium, which consisted of Advanced DMEM/F-12 medium (Invitrogen) supplemented with GlutaMAX-I (Invitrogen), HEPES (Invitrogen), Penicillin-Streptomycin (Invitrogen), B-27 and N-2 supplement (Invitrogen), N-Acetylcysteine (Sigma-Aldrich), Valproic acid (Sigma-Aldrich), Recombinant murine EGF and Noggin (Peprotech), Afamin/Wnt3a CM (MBL Life Science), Chir99021 (R&D Systems), Recombinant human FGF10 (Peprotech), Y-27632 and SB431542 (R&D Systems), R-Spondin1 conditioned medium (generated from Cultrex HA-R-Spondin1-Fc 293T cell line; R&D Systems). The organoids were cultured for 5 to 7 days and passaged using TrypLE Express (Invitrogen). In the indicated experiments, the aged gastric organoids were cultured with or without 2 μg/mL recombinant Dickkopf3 (Dkk3; Peprotech) in the medium that lacked Wnt3a, R-Spondin1, and Chir99021 (WRC− medium).

### Evaluation of Organoid Formation and Cell Viability Assay

Gastric organoids were taken apart by TrypLE Express, and cells were suspended in Matrigel. A total of 500 cells were plated per well of 96-well microplates. Organoids, more than 50 μm in diameter, were counted on days 0, 2, and 4. In addition, cellular proliferation was measured using CellTiter-Glo three-dimensional cell viability assay (Promega) on day 4.

### T-cell Factor Reporter Assay

Gastric organoids were dismantled by TrypLE Express. A total of 2.5 × 10^5^ cells were transfected with TOPFlash plasmid (Merck-Millipore) together with pRL-TK renilla control vector (Promega) using 4D-Nucleofector system (Lonza). Transfected cells were suspended in Matrigel and plated onto a 48-well multiple-well plate and incubated for 72 hours. The cells were then lysed, and luciferase activity was measured using Dual luciferase reporter assay kit (Promega).

### Microarray Analysis

Total RNA was extracted from young and aged gastric organoids using TRIzol reagent (Invitrogen), and 1 μg of RNA was utilized for Clariom S mouse array (Applied Biosystems). The acquired data were analyzed using gene set enrichment analysis (GSEA) software (Broad Institute).

### Quantitative Real-time Reverse Transcription PCR

cDNA was generated from 1 μg of RNA extracted from the organoids using SuperScript III First-strand synthesis system (Invitrogen). cDNA was then used as a template for quantitative real-time reverse transcription PCR (Q-PCR; StepOnePlus Real-time PCR systems; Applied Biosystems). The primer sets used for Q-PCR are listed in Table 2.

**TABLE 2:** Primer sets used for Q-PCR in this study

Primer name	Assay ID (ABI)	Primer name	Assay ID (ABI)
*Actb*	Mm02619580_g1	*Lgr5*	Mm00438890_m1
*Apc*	Mm00545872_m1	*Il6*	Mm010446190_m1
*Axin1*	Mm01299060_m1	*Lrp5*	Mm01227476_m1
*Axin2*	Mm00443610_m1	*Lrp6*	Mm00999795_m1
*Bcl9*	Mm01265706_m1	*Mki67*	Mm01278617_m1
*Ccnb1*	Mm03053893_gH	*Myc*	Mm00487804_m1
*Ccnd1*	Mm00432359_m1	*Rnf43*	Mm00552558_m1
*Cd44*	Mm01277163_m1	*Sfrp1*	Mm00489161_m1
*Cdk1*	Mm00772472_m1	*Sfrp2*	Mm01213947_m1
*Cdkn1a*	Mm04205640_g1	*Sfrp5*	Mm01194236_m1
*Cdkn2a*	Mm00494449_m1	*Tbx3*	Mm01195726_m1
*Cxcl1*	Mm04207460_m1	*Tnf*	Mm00443258_m1
*Dkk1*	Mm00438422_m1	*Tnfrsf19*	Mm00443506_m1
*Dkk3*	Mm00443800_m1	*Trp53*	Mm01731290_g1
*Fzd1*	Mm00445405_s1	*Wnt2b*	Mm00437330_m1
*Fzd2*	Mm02524776_s1	*Wnt3*	Mm00437336_m1
*Fzd7*	Mm00433409_s1	*Wnt3a*	Mm00437337_m1
*Gsk3b*	Mm00444911_m1	*Znrf3*	Mm01191453_m1

### Immunofluorescence

Gastric organoids were fixed in 4% paraformaldehyde. Antibody against the protein of interest was added to the organoids after blocking with 1% normal goat serum (Nichirei). After 24 hours of incubation at 4°C, an appropriate secondary antibody was added. The organoids were mounted with ProLong Diamond mountant with DAPI (Invitrogen) and observed with C2si confocal laser microscope (Nikon Instruments).

### Senescence-associated β-galactosidase Assay

After two passages, gastric organoids were cultured for 5 days. Following the addition of Bafilomycin A1 (Sigma-Aldrich) to the culture, the organoids were fixed with 4% paraformaldehyde. Cells containing senescence-associated β-galactosidase (SABG) were stained using SPiDER-βGal (Dojindo Laboratories) and mounted with ProLong Diamond mountant with DAPI. SABG positive cells were counted in three randomly selected microscopic fields.

### TBX3 Overexpression Study

AGS and HEK cells were transfected with either TBX3 human tagged ORF clone (Origene) or pCMV6-AC-DDK empty vector (Origene) using TransIT-X2 dynamic delivery system (TaKaRa Bio). After 48 hours of incubation, the transfected cells were applied for succeeding experiments. A total of 2 × 10^3^ cells were plated per well of 96-well plates. Cellular proliferation was evaluated with CellTiter 96 aqueous one solution cell proliferation assay (Promega) at 96 hours. On the other hand, the transfected cells were plated onto a 48-well plate to evaluate SABG using SPiDER-βGal.

### Western Blot Analysis

Total protein was extracted from AGS and HEK cells transfected with either TBX3-expressing plasmid or an empty vector, using a lysis buffer consisting of 0.05 mol/L Tris·HCl (pH 7.5), 0.15 mol/L NaCl, 1% Triton X-100, 1% dithiothreitol, and proteinase inhibitor cocktail tablet (Complete mini; Roche Diagnostics). The extracted protein was supplemented with Laemmli sample buffer (Bio-Rad), boiled, and electrophoresed on 10% Tris-glycine polyacrylamide gel (Bio-Rad) and transferred to polyvinylidene difluoride membrane (Immobilon P; Merck-Millipore). After blocking with ECL prime blocking agent (GE Healthcare), the membrane was incubated with a primary antibody against the protein of interest, followed by incubation with the appropriate secondary antibody. Protein bands were detected with ECL advance reagent (GE Healthcare) and visualized using ChemiDoc MP imaging system (Bio-Rad).

### Methylation-specific PCR

Genomic DNA was extracted from gastric organoids using DNeasy blood & tissue kit (Qiagen). The extracted DNA was subjected to bisulfite conversion and then utilized as the template for PCR. Epitaq HS (TaKaRa Bio) was used for amplification of the region containing CpG island in *Dkk3* promoter, and the primer pairs MF1 and MR1, or UF1 and UR1 were used for the detection of methylated and unmethylated CpG, respectively (Table 3). The PCR product was electrophoresed on 1% agarose gel containing ethidium bromide.

**TABLE 3:** Primer sets used for methylation-specific PCR in this study

Primer name	Sequence
MF1	5′-GAATAAATATGTAGCGGTTCGG-3′
MR1	5′-TCCTAAAAATAATTAAAAACTAAACCCG-3′
UF1	5′-ATAAATATGTAGTGGTTTGGGG-3′
UR1	5′-AAAAATAATTAAAAACTAAACCCAACTCC-3′

### IHC

Twenty-nine biopsy specimens obtained by esophagogastroduodenoscopy and 11 gastric intramucosal cancer specimens obtained by endoscopic submucosal dissection were utilized for IHC under the permission of the ethics committee of Tohoku University Graduate School of Medicine (Miyagi, Japan). Written informed consent was obtained from these patients. IHC for TBX3, Ki67, and DKK3 was performed as described previously (17). Briefly, serial sections cut from paraffin blocks were dewaxed, and endogenous peroxidase activity was blocked by hydrogen peroxidase solution. The sections were then autoclaved in 10 mmol/L citrate buffer, supplied with primary antibody against the protein of interest, and incubated overnight at 4°C. The sections were then applied with secondary antibody and visualized using DAB+ substrate chromogen system (Dako Cytomation). The intensity of DKK3 staining was evaluated as negative:0, weak:1, intermediate:2, and strong:3, according to a precedent report (18).

### Study Approval

The procedures listed above were approved by the Institutional Animal Care and Use Committee at Tohoku University (Miyagi, Japan). All studies were conducted in accordance with recognized ethical guidelines (including the Declaration of Helsinki, CIOMS, Belmont Report, U.S. Common Rule) and approved by the Institutional Review Board of Tohoku University Graduate School of Medicine (Miyagi, Japan).

### Statistical Analysis

Statistical analysis was performed using Excel 2016 (Microsoft), R (The R Foundation for Statistical Computing), and EZR (Jichi Medical University Saitama Medical Center, Saitama, Japan). Student *t* test was used to evaluate the significance of the differences between the two groups, and a value of *P* < 0.05 was considered statistically significant. Bonferroni procedure was employed in the case where three groups were compared, and *P* < 0.017 was considered as statistically significant. Pearson correlation coefficient was used for product-moment correlation analysis, and a value of *P* < 0.05 was regarded as statistically significant. Results are shown as the mean ± standard error.

### Data Availability

The data acquired from microarray analysis were deposited in the Gene Expression Omnibus database (https://www.ncbi.nlm.nih.gov/geo/; accession number: GSE185680). Data supporting the findings of this study will be available from the corresponding author upon a reasonable request.

## Results

### Cellular Proliferation is Enhanced in Aged Gastric Organoids and is Dependent on Wnt/β-catenin Signaling

**FIGURE 1:**
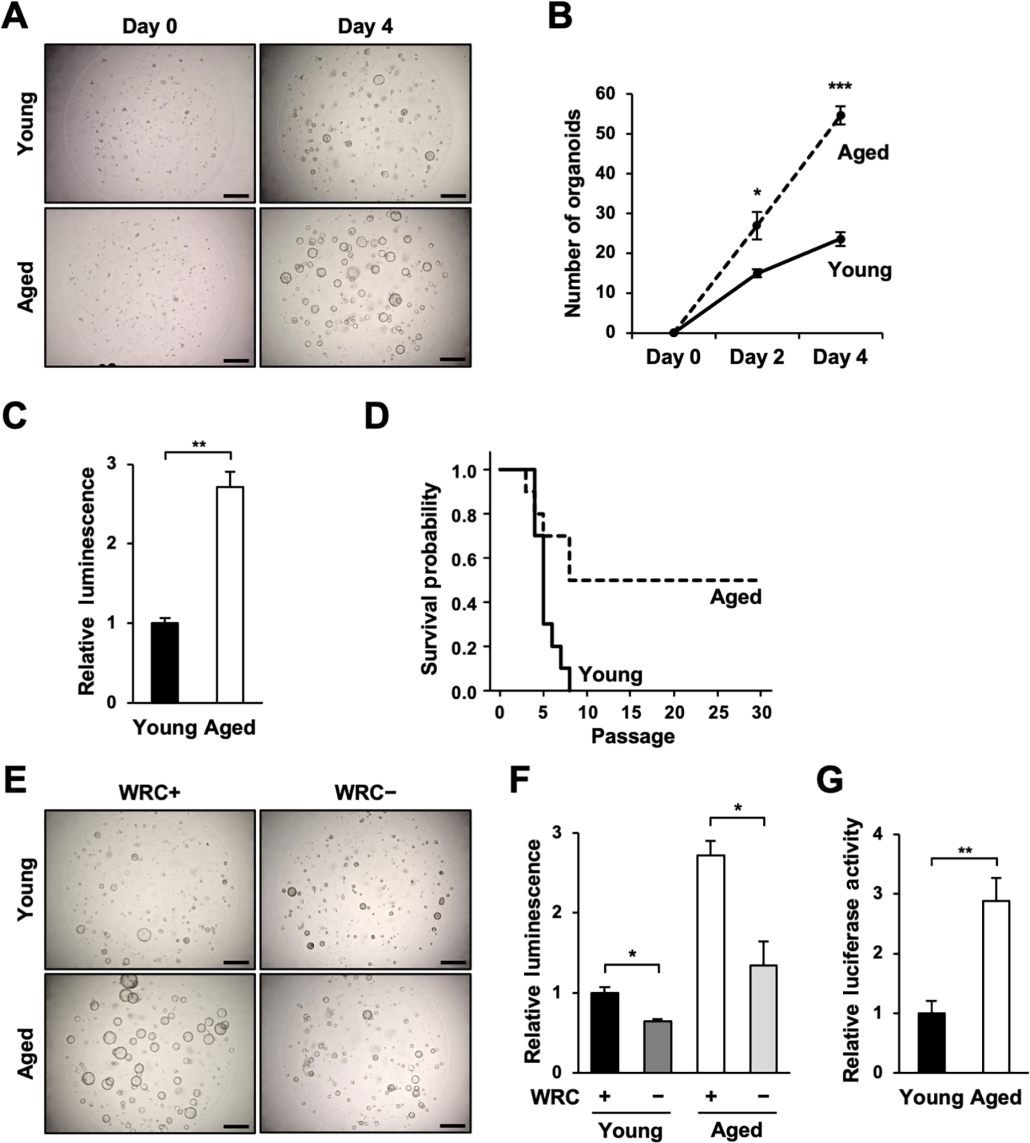
Aged gastric organoids exhibit increased cellular proliferation and enhanced Wnt/β-catenin signaling. **A,** Representative images of young and aged gastric organoids on days 0 and 4. *Scale bar*: 500 μm. **B,** The numbers of young and aged gastric organoids larger than 50 μm in diameter on days 0, 2, and 4 (*n* = 3). **C,** Cell viability assay of young and aged gastric organoids performed on day 4. **D,** Survival of the young and aged gastric organoids (*n* = 3). **E,** Representative images of young and aged gastric organoids cultured in the presence (WRC+) and the absence (WRC−) of Wnt3a, R-Spondin1, and Chir99021. *Scale bar*: 500 μm. **F,** Cell viability assay of young and aged gastric organoids cultured in WRC+ and WRC− medium performed on day 4 (*n* = 3). **G,** TCF reporter activity of young and aged gastric organoids (*n* = 3). n.s., not significant; *, *P* < 0.05; **, *P* < 0.01; ***, *P* < 0.001.

Gastric organoids were generated from 10 young (2–4 months of age) and 10 aged (>18 months of age) mice. After the second passage, we compared the numbers of the formed organoids and their cellular proliferation. We found that the number of gastric organoids formed from aged mice was significantly greater than that from young mice (26.83 ± 3.48 vs. 14.93 ± 1.03 on day 2 and 54.60 ± 2.28 vs. 23.50 ± 1.72 on day 4, respectively; Fig. 1A and B). Cell viability assay of the same organoids revealed that cellular proliferation was enhanced in aged gastric organoids compared to young gastric organoids (2.71 ± 0.19 folds; Fig. 1C). Further passaging of the organoids unveiled that while we were only able to passage young gastric organoids for eight passages or less, we were able to passage more than 30 passages in 5 of 10 aged gastric organoids (Fig. 1D).

To evaluate the implications of Wnt/β-catenin signaling on the formation and proliferation of the organoids, we cultured young and aged gastric organoids in the presence (WRC+) and the absence (WRC−) of Wnt3a, R-Spondin1, and Chir99021. The organoid formation and cellular proliferation were reduced in both young and aged gastric organoids when cultured in WRC− medium, suggesting that the enhanced organoid formation and cellular proliferation of aged gastric organoids depended on Wnt/β-catenin signaling (0.64 ± 0.03 and 0.49 ± 0.11 folds in young and aged gastric organoids, respectively; Fig. 1E and F). Indeed, Wnt/β-catenin signaling in aged gastric organoids was significantly enhanced compared with young gastric organoids as measured by T-cell factor (TCF) reporter assay (2.89 ± 0.38 folds; Fig. 1G). Taken together, the above studies suggested that Wnt/β-catenin signaling is intensified in aged gastric organoids and consequently accelerate cellular proliferation and organoid formation.

### Aged Gastric Organoids Evade Cellular Senescence Through Enhanced G_2_–M Transition

**FIGURE 2:**
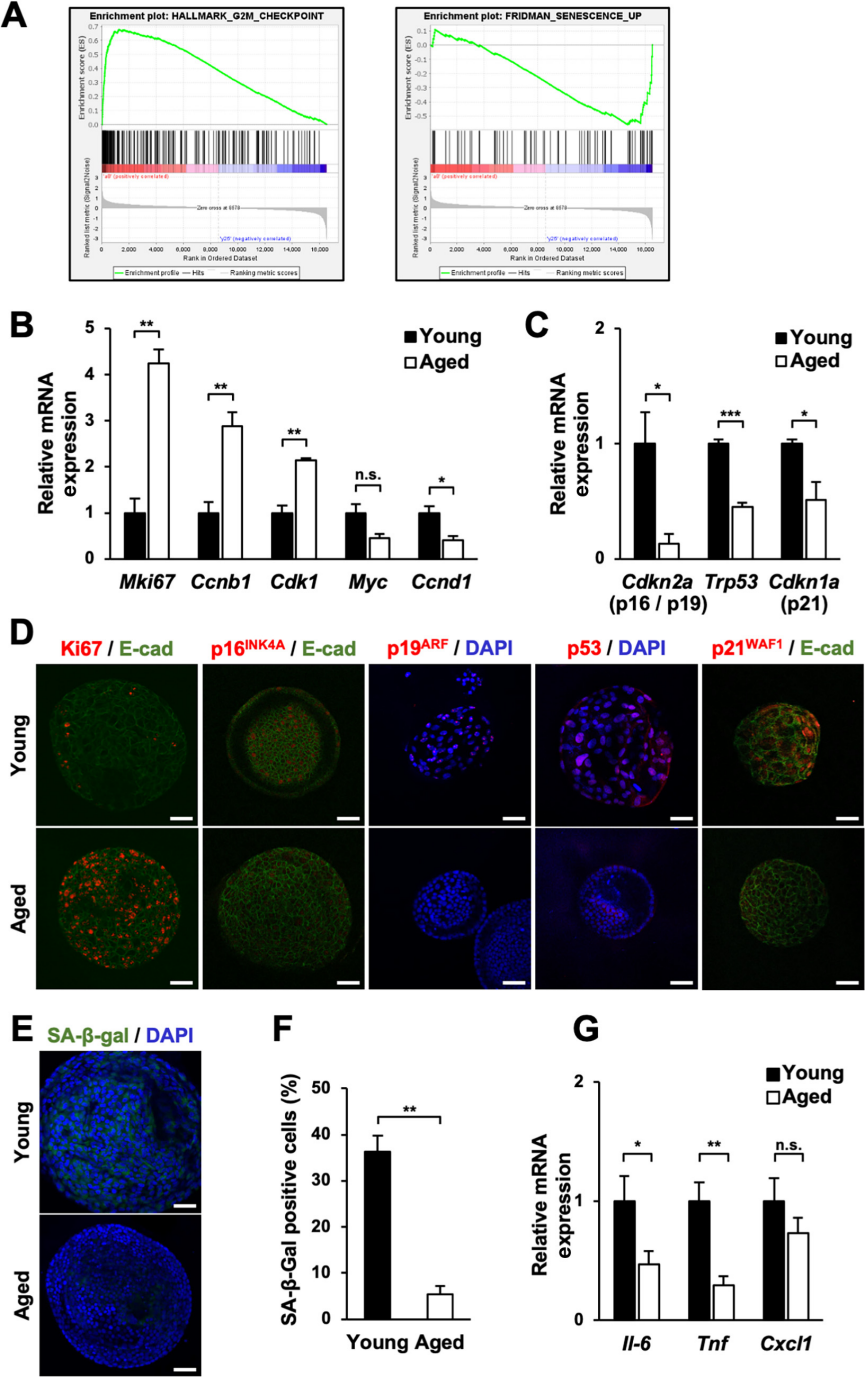
Cellular senescence is suppressed in aged gastric organoids. **A,** GSEA for (left) G_2_–M checkpoint-related genes and (right) senescence-related genes. **B,** The expression of genes related to cellular proliferation and cell cycle in young and aged gastric organoids (*n* = 3). **C,** The expression of senescence-inducing genes in young and aged gastric organoids (*n* = 3). **D,** Immunofluorescence for genes related to cellular proliferation and senescence in young and aged gastric organoids (*n* = 5). E-cad: E-cadherin. **E,** Representative images of SABG assay for young and aged gastric organoids. *Scale bar*: 50 μm. **F,** The ratio of senescent cells in young and aged gastric organoids assessed by SABG assay (*n* = 3). **G,** The expression of SASP-related genes in young and aged gastric organoids (*n* = 3). n.s., not significant; *, *P* < 0.05; **, *P* < 0.01; ***, *P* < 0.001.

To determine the factors that influenced cellular proliferation and organoid formation downstream of Wnt/β-catenin signaling in young and aged gastric organoids, we extracted the total RNA from young and aged gastric organoids and performed a microarray analysis. GSEA of the acquired microarray data revealed that aged gastric organoids positively correlated with G_2_–M checkpoint-related gene sets (HALLMARK_G2M_CHECKPOINT: NES = 2.79, Nominal *P* value = 0.0, FDR *Q* value = 0.0; Fig. 2A, left). At the same time, they showed a negative correlation with senescence-related gene sets (FRIDMAN_SENESCENCE_UP: NES = −1.88, Nominal *P* value = 0.0, FDR *Q* value = 0.01; Fig. 2A, right). Q-PCR for the cell cycle–related genes revealed that while the expression of genes related to the G_1_–S phase such as *Myc* and *Ccnd1* was suppressed, the expression of G_2_–M-phase–related genes such as *Ccnb1* and *Cdk1* was increased in aged gastric organoids (Fig. 2B). In addition, the expression of *Mki67*, which encodes the marker of proliferation Ki67, was significantly enhanced in aged gastric organoids (4.24 ± 0.30 folds; Fig. 2B). Furthermore, analyses of cellular senescence-inducing genes revealed that the expression of *Cdkn2a*, *Trp53*, and *Cdkn1a* was significantly reduced in aged gastric organoids (0.13 ± 0.09, 0.45 ± 0.31, and 0.51 ± 0.16 folds, respectively; Fig. 2C). Immunofluorescence confirmed these results, which showed increased expression of Ki67 and reduced expression of p16^INK4A^, p19^ARF^, p53, and p21^WAF1^ in aged gastric organoids (Fig. 2D).

Because the expression of senescence-related genes was reduced in aged gastric organoids, we then examined whether cellular senescence was suppressed in aged gastric organoids. SABG assay of young and aged gastric organoids, after two passages, unveiled that aged gastric organoids contained fewer senescent cells compared with young gastric organoids (5.33% ± 1.86% and 36.33% ± 3.48%, respectively; Fig. 2E and F). This finding obtained from SABG assay was confirmed by the evaluation of senescence-associated secretory phenotype (SASP)-related genes. The expression of *Il-6* and *Tnf* was significantly reduced, and the expression of *Cxcl1* tended to be lower in aged gastric organoids (0.47 ± 0.11, 0.30 ± 0.08, and 0.73 ± 0.13 folds, respectively; Fig. 2G). These data indicated that cellular senescence is suppressed in aged gastric organoids.

### Aged Gastric Organoids Express Wnt/β-catenin Signaling Target Gene T-box3

**FIGURE 3:**
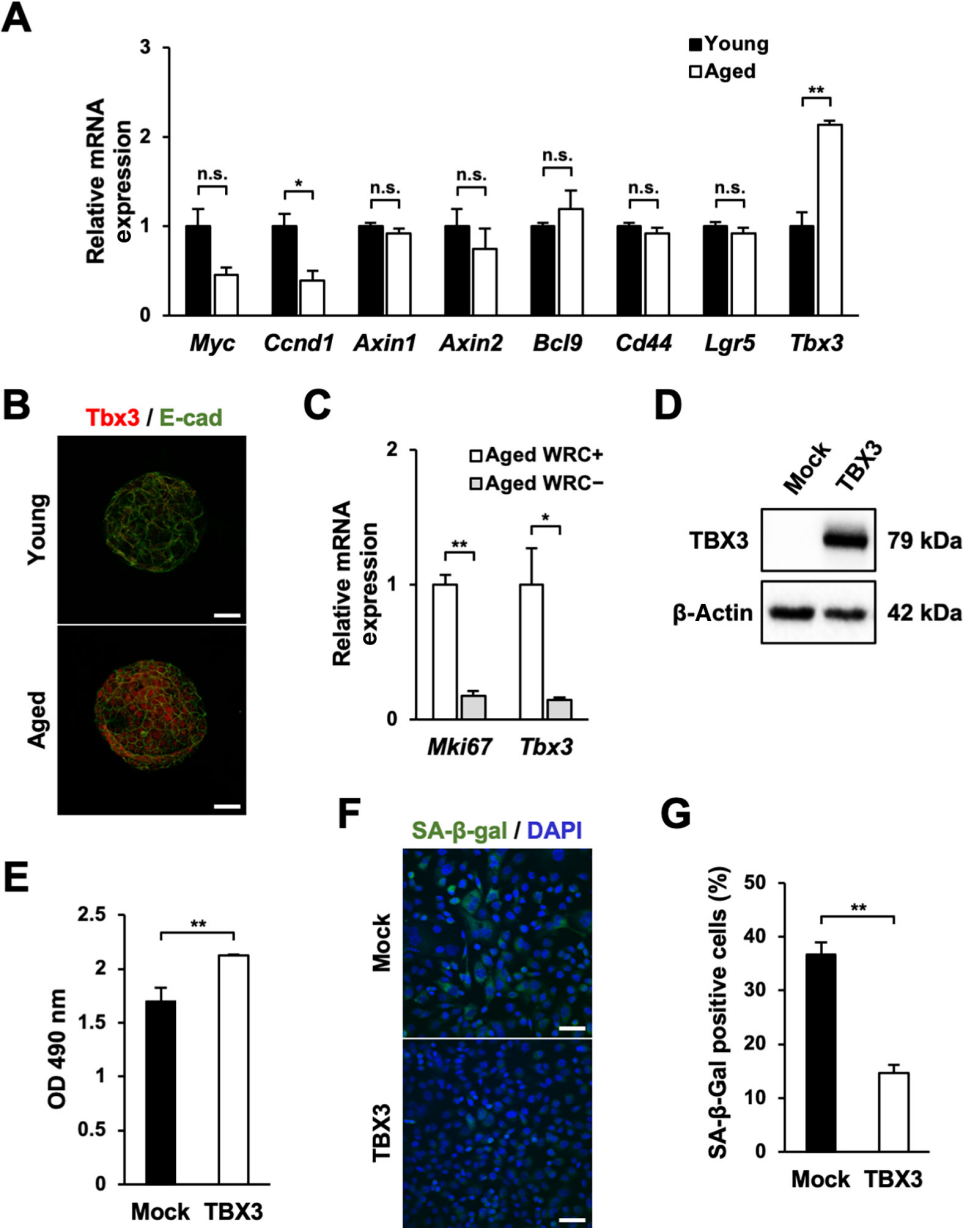
Aged gastric organoids express Tbx3. **A,** The expression of Wnt target genes in young and aged gastric organoids (*n* = 3). **B,** Immunofluorescence for Tbx3 in young and aged gastric organoids (*n* = 5). E-cad: E-cadherin. **C,***Mki67* and *Tbx3* expression in the presence (WRC+) and the absence (WRC−) of Wnt3a, R-Spondin1, and Chir99021 in aged organoids (*n* = 3). **D,** Western blot analysis for TBX3 and β-actin of AGS cells transfected with empty vector or TBX3-expressing plasmid. **E,** MTS assay for TBX3-overexpressing AGS cells (*n* = 3). **F,** Representative images of SABG assay for TBX3-overexpressing AGS cells. *Scale bar*: 50 μm. **G,** The ratio of senescent cells in TBX3-overexpressing AGS cells assessed by SABG assay (*n* = 3). n.s., not significant; *, *P* < 0.05; **, *P* < 0.01; ***, *P* < 0.001.

The studies above demonstrated that cellular senescence of aged organoids is suppressed, resulting in increased cellular proliferation and that this mechanism is dependent on Wnt/β-catenin signaling. Therefore, we surmised that the Wnt/β-catenin signaling target gene affected the cellular senescence-related genes and attempted to specify the responsible gene. Among the Wnt/β-catenin signaling target genes listed in Nusse's website (http://web.stanford.edu/group/nusselab/cgi-bin/wnt/), we checked the expression of eight genes (*Myc*, *Ccnd1*, *Axin1*, *Axin2*, *Bcl9*, *Cd44*, *Lgr5*, *Tbx3*) in our acquired microarray data. We found that the expression of transcription factor *Tbx3* was significantly increased in aged gastric organoids and this increasement was confirmed by Q-PCR (2.13 ± 0.05 folds; Fig. 3A). Because this transcription factor was reported to suppress cellular senescence (19, 20), we focused on this gene. Increased expression of T-box3 (Tbx3) in aged gastric organoids was further confirmed by immunofluorescence (Fig. 3B). However, this enhanced expression of Tbx3 was obliterated when the aged gastric organoids were cultured in the absence of Wnt3a, R-Spondin1, and Chir99021 (WRC− media; 0.14 ± 0.02 folds; Fig. 3C). Taken together, these studies suggested that the expression of Tbx3 in aged gastric organoids is enhanced in a Wnt/β-catenin signaling-dependent manner.

### Overexpression of TBX3 Accelerates Proliferation and Suppresses Cellular Senescence

From the above-mentioned studies, we confirmed that aged gastric organoids exhibited enhanced expression of Tbx3. In the succeeding study, we verified whether this transcription factor affected proliferation and cellular senescence. To this end, we transfected the TBX3-expressing plasmid into the AGS cell line (Fig. 3D). We were able to corroborate that overexpression of TBX3 led to accelerated proliferation as assessed by MTS assay (1.25 ± 0.01 folds; Fig. 3E). Furthermore, we found that the ratio of SABG-expressing cells was significantly lower in TBX3-overexpressing cells than control cells (14.67% ± 1.45% vs. 36.67% ± 2.33%, respectively; Fig. 3F and G). Similar results were obtained from TBX3-overexpressing HEK cells (Supplementary Fig. S1A–S1D). These results suggested that increased cellular proliferation and curbed cellular senescence seen in aged gastric organoids are induced by enhanced Tbx3 expression.

### The Expression of Dkk3 in Aged Gastric Organoids is Suppressed Because of Methylation of its CpG Island

**FIGURE 4:**
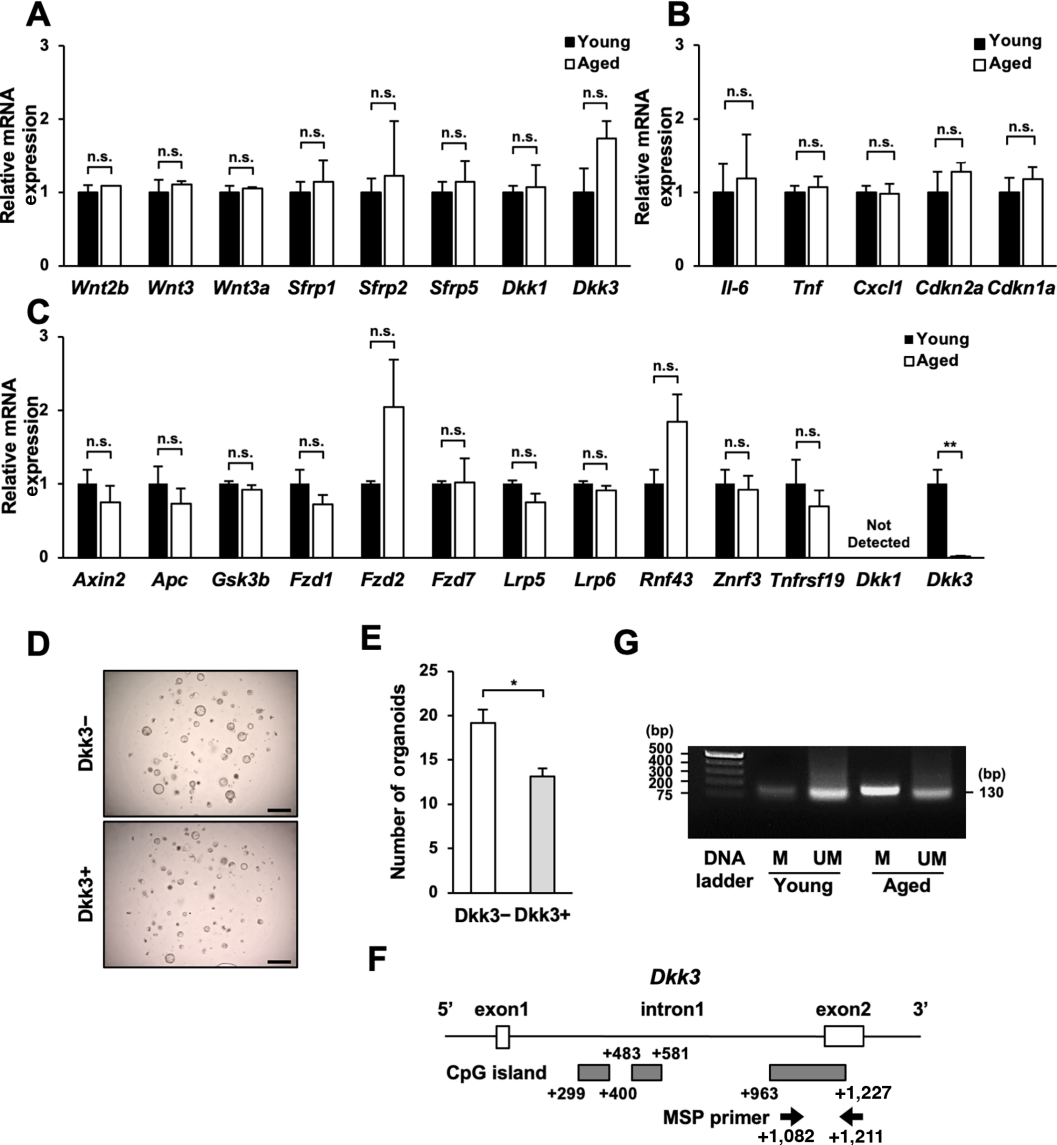
Negligible expression of Dkk3 in aged gastric organoids induces enhanced organoid-forming capacity. **A,** The expression of niche factors in the gastric mucosae of young and aged mice (*n* = 3). **B,** The expression of SASP-and senescence-related genes in the gastric mucosae of young and aged mice (*n* = 3). **C,** The expression of genes involved in Wnt/β-catenin signaling in young and aged gastric organoids (*n* = 3). **D,** Representative images of aged gastric organoids cultured in the presence and the absence of recombinant Dkk3 protein. *Scale bar*: 500 μm. **E,** The numbers of aged gastric organoids larger than 100 μm in diameter cultured in the presence and the absence of recombinant Dkk3 protein (*n* = 3). **F,** A schema showing the location of CpG islands and the primers designed for methylation-specific PCR. **G,** Methylation-specific PCR for genomic DNA extracted from young and aged gastric organoids (*n* = 3). n.s., not significant; *, *P* < 0.05; **, *P* < 0.01; ***, *P* < 0.001.

To identify the cause of enhanced Wnt/β-catenin signaling in aged gastric organoids, we focused on the factors that influenced this signaling pathway. At first, we appraised the expression of niche-related genes (*Wnt2b*, *Wnt3*, *Wnt3a*, *Sfrp1*, *Sfrp2*, *Sfrp5*, *Dkk1*, *Dkk3*) in the gastric mucosae of young and aged mice, but we were unable to detect any difference among them (Fig. 4A). In addition, we did not detect any difference in the expression of SASP-related and cellular senescence–related genes in the gastric mucosae of young and aged mice (*Il-6*, *Tnf*, *Cxcl1*, *Cdkn2a*, *Cdkn1a*; Fig. 4B). Then we assessed the expression of genes related to the Wnt/β-catenin signaling pathway (*Axin2*, *Apc*, *Gsk3b*, *Fzd1*, *Fzd2*, *Fzd7*, *Lrp5*, *Lrp6*, *Rnf43*, *Znrf3*, *Tnfrsf19*, *Dkk1*, *Dkk3*) in young and aged gastric organoids, and found that although young gastric organoids expressed *Dkk3*, the aged gastric organoids expressed extremely less amount of *Dkk3* compared with young gastric organoids (0.016 ± 0.005 folds; Fig. 4C). Dkk is a secreted protein that acts as a Wnt inhibitor (10) and is secreted not only from niche-related cells but also from the stem cells themselves (21). Hence, we figured that the Dkk3 expressed in the organoids could be affecting their Wnt/β-catenin signaling. To further assess the impact of Dkk3 on the organoid formation of aged gastric organoids, recombinant Dkk3 was added to the culture of aged gastric organoids, which resulted in significantly less organoid formation (0.69 ± 0.05 folds; Fig. 4D and E). These findings suggested that suppressed Dkk3 expression in aged gastric organoids contributed to its increased capability for organoid formation.

We then sought to identify the rationale behind the suppressed expression of Dkk3 in aged organoids. Because Dkk3 was previously reported to be suppressed through methylation (22) and it is well established that methylation of genes in the stomach occurs with age (23), we investigated whether *Dkk3* of aged gastric organoids was methylated. Search for CpG islands that alter the translation of *Dkk3* with MethPrimer 2.0 (http://www.urogene.org/methprimer2/) revealed three CpG islands in intron1 of the *Dkk3* gene (Fig. 4F). Among these islands, we paid attention to the CpG island between +963 and +1,227, which was the only CpG island that MethPrimer 2.0 was able to design primer pairs. Methylation-specific PCR revealed that this CpG island was indeed methylated in the genomic DNA of aged gastric organoids, whereas it was not methylated in that of young gastric organoids (Fig. 4G). Hence, these data implied that the expression of Dkk3 is suppressed in aged gastric organoids due to methylation of its CpG island.

### TBX3 is Expressed in Human Atrophic Gastritis and Gastric Cancer Tissues, and its Expression Increases with Age

**FIGURE 5:**
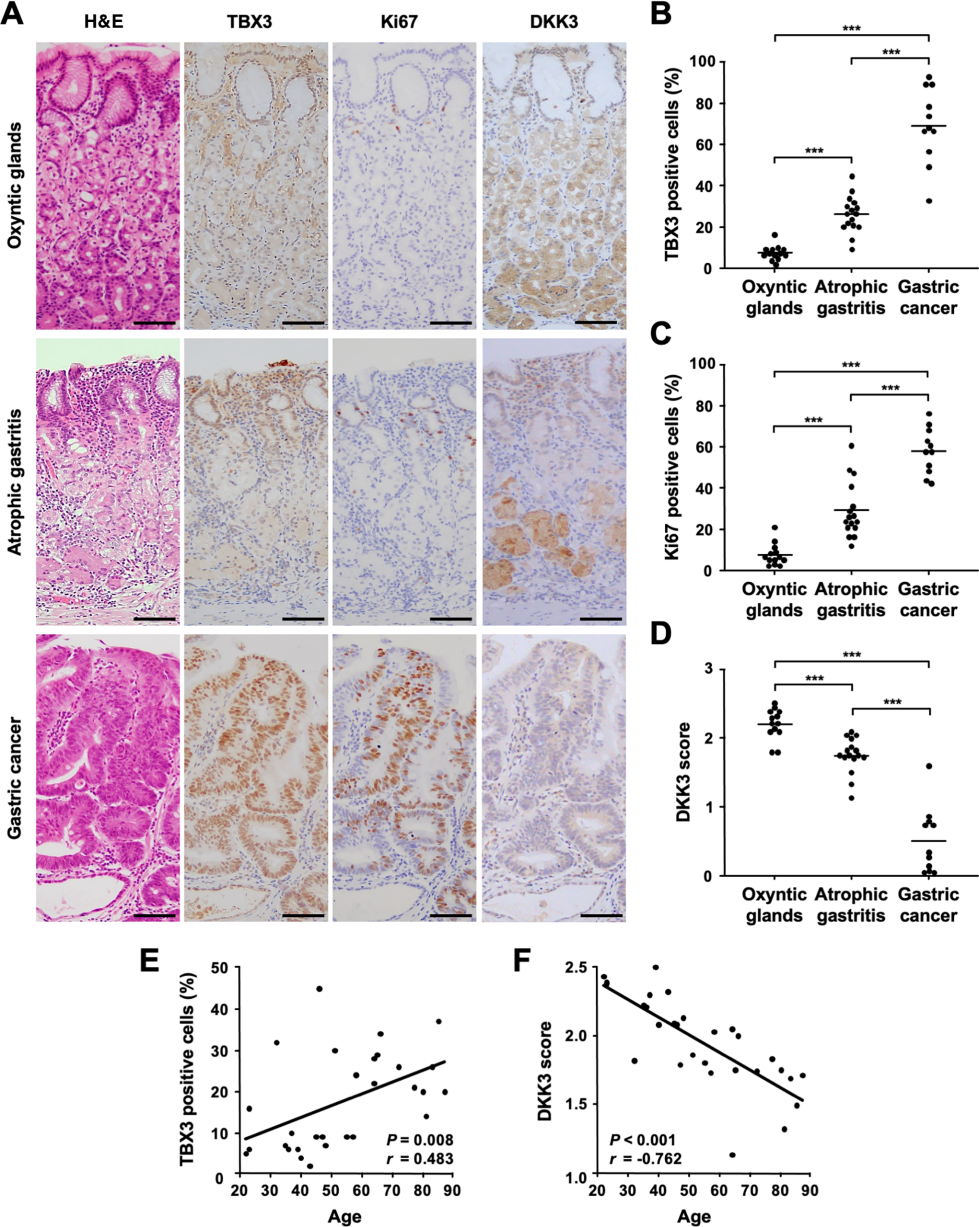
TBX3 is expressed in human atrophic gastritis and gastric cancer tissues. **A,** IHC for TBX3, Ki67, and DKK3 in human oxyntic glands, atrophic gastritis, and gastric cancers. *Scale bar*: 100 μm. TBX3 positivity (**B**), Ki67 positivity (**C**), and DKK3 score (**D**) in human oxyntic glands, atrophic gastritis, and gastric cancers (*n* = 40). **E,** Correlation of patients’ age and TBX3 positivity (*r* = 0.483, *P* = 0.008). **F,** Correlation of patients’ age and DKK3 score (*r* = −0.762, *P* < 0.001). ***, *P* < 0.001.

In the final sets of studies, we examined whether the findings obtained from young and aged gastric organoids applied to the human stomach. To this end, we evaluated the expression of TBX3, Ki67, and DKK3 in human gastric tissues utilizing IHC. The average ages of the oxyntic gland (*n* = 13), atrophic gastritis (*n* = 16), and gastric cancer cases (*n* = 11) were 37.9 ± 2.9, 66.8 ± 3.9, and 74.1 ± 3.0 years old, respectively. In the oxyntic gland cases, TBX3 was expressed in the isthmus and the mucosal surface cells (7.38% ± 0.95%; Fig. 5A and B). In the atrophic gastritis cases, it was also expressed in the isthmus, and due to the elongation of the isthmus, there were more TBX3 positive cells in atrophic gastritis tissues compared with the oxyntic gland tissues (26.06% ± 2.20%; Fig. 5A and B). On the other hand, most of the gastric cancer cells expressed TBX3 (69.18% ± 5.53%; Fig. 5A and B). A similar tendency was observed with the Ki67 expression (7.62% ± 1.47% in oxyntic glands, 29.25% ± 3.38% in atrophic gastritis, 58.18% ± 3.34% in gastric cancer; Fig. 5A–C). On the other hand, DKK3 showed a reciprocal expression to TBX3 (2.20 ± 0.06 in oxyntic glands, 1.75 ± 0.06 in atrophic gastritis, 0.52 ± 0.14 in gastric cancer; Fig. 5A–D).

Because our aim of this study was to elucidate the implication of aging on carcinogenesis, we then assessed the correlation of age with TBX3 and DKK3 expression in patients without cancer. We found a positive correlation between patients’ age and TBX3 positive ratio (Fig. 5E; *r* = 0.483, *P* = 0.008) and a negative correlation between patients’ age and DKK3 expression (Fig. 5F; *r* = −0.762, *P* < 0.001). These results suggested that TBX3 expression in the gastric mucosa increased with age and that the increment of TBX3 expression could be related to gastric carcinogenesis.

## Discussion

In this study, we discovered the emergence of vigorously proliferating organoids in those established not from young but from aged murine stomach. Wnt/β-catenin signaling in these organoids was augmented because of their reduced Dkk3 expression resulting from methylation of its CpG island. The intensified Wnt/β-catenin signaling led to enhanced expression of Tbx3 in these aged organoids, which caused enhanced G_2_–M transition and the avoidance of cellular senescence, resulting in increased cellular proliferation. This TBX3 expression was observed in human atrophic gastritis and its expression was even more enhanced in gastric cancer tissues. Furthermore, TBX3 positive ratio in the gastric tissue exhibited positive correlation with patients’ age. These findings suggested that this Dkk3-Wnt-Tbx3 pathway identified in aged gastric organoids is involved in aging-related gastric carcinogenesis.

Wnt/β-catenin signaling is known to play an essential role in the differentiation and proliferation of stem cells (10). Regarding the investigation of aging, Nalapareddy and colleagues reported that aged murine small intestine expressed decreased level of Wnt3a, and declined proliferation of aged murine small intestinal organoids was resurrected by adding Wnt3a to the organoid culture medium (16). On the other hand, Pentinmikko and colleagues reported that aged murine small intestinal organoids had reduced organoid-forming capacity and that this was due to the production of Notum, an extracellular Wnt inhibitor, from aged Paneth cells (24). Therefore, at the inception of this study, we expected to see decreased Wnt/β-catenin signaling and cellular proliferation in aged gastric organoids. However, contrary to our expectations, aged gastric organoids in our study showed greater capability to form organoids and were able to passage longer compared with young gastric organoids. Furthermore, although we did not detect any difference in the expression of Wnt family genes between aged and young stomach, aged gastric organoids showed enhanced Wnt/β-catenin signaling. Possible explanations for this discrepancy between our study and the prior reports from Nalapareddy and Pentinmikko could be in that their studies were based on small intestine, the organ with limited potential for developing cancers (25).

In the current study, aged gastric organoids showed enhanced Wnt/β-catenin signaling and possessed greater capability to proliferate and to form organoids compared with young gastric organoids, and this advantage was lost in the absence of Wnt3a, R-Spondin1, and Chir99021. This clearly indicated that the cellular proliferation and organoid formation is dependent on Wnt/β-catenin signaling. In the living body, Wnt and R-Spondin are considered to be derived from stromal cells (26). On the other hand, in the organoid culture system where there are no stromal cells, these niche factors are artificially added to the culture, enabling us to investigate the role they play in gastric homeostasis.

One mechanism responsible for the augmented Wnt/β-catenin signaling in aged gastric organoids that we identified was the reduced expression of Dkk3. Dkk3 is a secreted protein that hampers Wnt/β-catenin signaling by binding to low-density lipoprotein receptor–related protein (LRP) 5/6 (10), one of the receptors of Wnt/β-catenin signaling. In the stomach, its expression has been identified in epithelial cells, and it has been reported to participate in the regulation of Wnt/β-catenin signaling of the gastric epithelial stem cells (26). We found that Dkk3 expression in aged gastric organoids was reduced because of DNA methylation and this epigenetic alteration was not observed in young gastric organoids. Considering the previous report which showed that DNA methylation found in the intestinal organoids reflects that of the original intestinal mucosa (27), we surmised that the aberrant DNA methylation of *Dkk3* seen in aged gastric organoids reflects that of gastric stem cells in the aged gastric mucosa and that aging gastric mucosa gave rise to *Dkk3*-methylated cells within the gastric stem cells. These cells had increased ability to proliferate due to enhanced Wnt/β-catenin signaling, hence, the organoids formed from these cells predominated over other organoids. Dkk3 has been regarded as a tumor suppressor (28, 29), and the methylation of *Dkk3* has been reported to be associated with poor prognosis in advanced gastric cancers (22) and breast cancers (30). Considering that the methylation of critical genes, such as cell cycle–related genes and tumor suppressor genes, has been reported to initiate the formation of neoplasm (31), and considering that the methylation of *DKK3* has been reported to be involved in the initiation of hepatocellular carcinomas (32), the emergence and the predominance of *Dkk3*-methylated gastric stem cells could be involved in the initiation of aging-related gastric carcinogenesis.

Regarding the source of Dkk3, we did not see any difference in the expression of *Dkk3* between aged and young gastric mucosae. However, we were able to detect significant difference in *Dkk3* expression between the aged and young gastric organoids, which suggested that the source of Dkk3 influencing the growth was the gastric stem cells themselves. A previous report which showed that Dkk3 produced from interfollicular epidermal stem cells operate in an autocrine manner to maintain stem cells’ self-renewal (21) reinforce our theory.

The intensified Wnt/β-catenin signaling seen in the aged gastric organoids resulting from decreased Dkk3 expression led to induction of the transcription factor Tbx3, which is a Wnt/β-catenin signaling target gene involved in the survival of liver cancer cells (33). TBX3 was first reported to be implicated in ulnar-mammary syndrome, a rare pleiotropic disorder with defects of the upper limb, tooth, and genitalia, and hypoplasia of the mammary and apocrine glands (34). It has also been reported to be related to the evasion of cellular senescence by suppressing the expression of cell cycle–related genes such as p19^ARF^, p53, and p21^WAF1^ (19). In the current study, we found that aged gastric organoids expressed a high amount of Tbx3 and less p19^ARF^, p53, and p21^WAF1^ compared with young gastric organoids. In addition, we were able to detect increased cellular proliferation and decreased cellular senescence in TBX3-overexpressing cells through mechanistic overexpression studies. These results indicated that TBX3, expressed in aged gastric organoids, suppressed cellular senescence.

The aged gastric organoids expressed less p19^ARF^, p53, and p21^WAF1^, and more Ccnb1 and Cdk1 than young gastric organoids, suggesting that cellular senescence was evaded because of enhanced G_2_–M transition. In addition, elevated Ccnb1 levels, as was seen in the aged gastric organoids of the current study, have been frequently seen in advanced gastric cancers (35) and breast cancers (36). This, together with the induced Tbx3 and suppressed Dkk3, suggested that the aged gastric organoids in the current study possibly possessed the potential to develop gastric cancer.

Regarding the role of TBX3 in cancers, the expression of TBX3 has been reported to be enhanced in advanced gastric cancers (37) and breast cancers (38) and contributed to the progression of these diseases. Moreover, it has also been reported to play a pivotal role in the early stage of carcinogenesis in liver cancers (33). In the current study, we assessed the expression of TBX3 in human gastric tissues and found that the expression of TBX3 was increased in precancerous atrophic gastritis and even more increased in gastric cancer tissues. Furthermore, expression of TBX3 in the gastric epithelial cells increased according to patients’ age. On the contrary, expression of DKK3 decreased as the severity of gastric lesions and patients’ age increased, which reinforces our findings with TBX3. Together with the prior reports, these findings suggested that enhanced expression of TBX3 in gastric epithelial cells could play an essential role in the early stage of age-related gastric carcinogenesis.

In the current study, we found that the aged gastric organoids were able to form more organoids and proliferate more vigorously than the young gastric organoids and that the aged gastric organoids exhibited less cellular senescence. However, the evaluation of SASP- and senescence-related genes of the gastric mucosae which the organoids originated from, did not show any difference. This finding suggests that *Dkk3*-methylated gastric epithelial cells that rise in the gastric mucosae of aged mice, are minority in the physiologic state, but they expand in the organoid culture due to abundant Wnt/β-catenin signaling stimulating factors.

The strength of the current study lies in that by utilizing the organoid culturing system, we were able to show one possible mechanism for aging-related carcinogenesis, which is the overwhelming expansion of aged gastric stem cells with methylated *Dkk3*. However, our limitation lies in that we did not investigate mutations, which is another aging-related change which contributes to carcinogenesis (39).

In conclusion, we determined that organoids established from the stomachs of aged mice showed accelerated proliferation and enhanced survival due to evasion of cellular senescence. This mechanism might be involved in aging-related gastric carcinogenesis.

## Supplementary Material

Supplementary Figure 1 Supplementary Figure 1. TBX3 suppresses senescence and enhances proliferation. (A) Western blot for TBX3 and b-actin of HEK cells transfected with empty vector or TBX3-expressing plasmid. (B) MTS assay for TBX3- overexpressing HEK cells (n=3). (C) Representative images of SABG assay for TBX3-overexpressing HEK cells. Scale bar: 50μm. (D) The ratio of senescent cells in TBX3-overexpressing HEK cells assessed by SABG assay (n=3). **:P<0.01.
