# Supplementary material for: Suppressed Cellular Senescence Mediated by T-box3 in Aged Gastric Epithelial Cells may Contribute to Aging-related Carcinogenesis
Source: Cancer Res Commun. 2022 Aug 8;2(8):772–83. doi: 10.1158/2767-9764.CRC-22-0084 (PMC10010334; doi:10.1158/2767-9764.CRC-22-0084)
Supplement: Supplementary Figure 1 — TBX3 suppresses senescence and enhances proliferation. (A) Western blot for TBX3 and b-actin of HEK cells transfected with empty vector or TBX3-expressing plasmid. (B) MTS assay for TBX3- overexpressing HEK cells (n=3). (C) Representative images of SABG assay for TBX3-overexpressing HEK cells. Scale bar: 50μm. (D) The ratio of senescent cells in TBX3-overexpressing HEK cells assessed by SABG assay (n=3). **:P<0.01. [file crc-22-0084-s01.pdf]

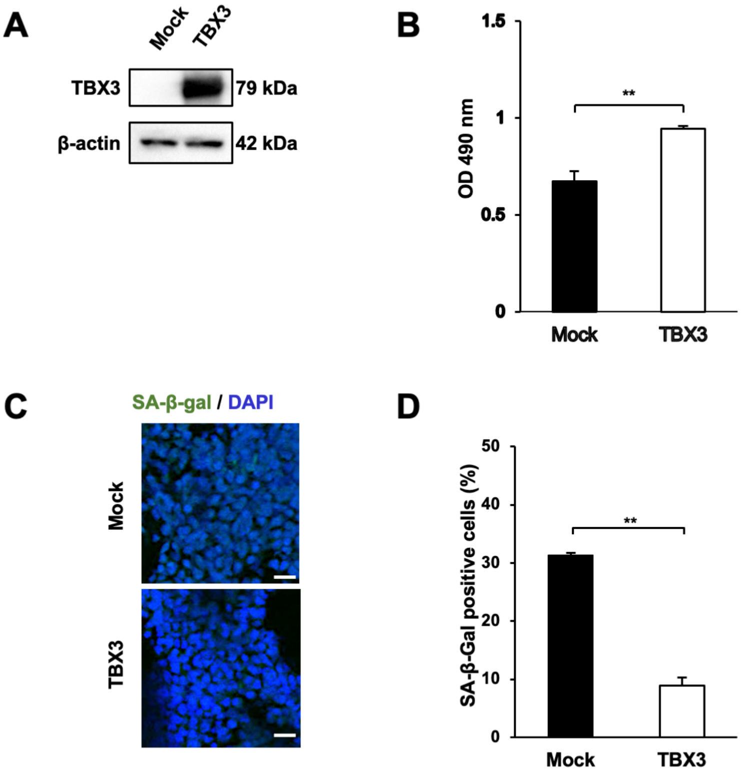

Supplementary Figure 1. TBX3 suppresses senescence and enhances proliferation. (*A*) Western blot for TBX3 and β-actin of HEK cells transfected with empty vector or TBX3-expressing plasmid. (*B*) MTS assay for TBX3-overexpressing HEK cells (n=3). (*C*) Representative images of SABG assay for TBX3-overexpressing HEK cells. *Scale bar*: 50μm. (*D*) The ratio of senescent cells in TBX3-overexpressing HEK cells assessed by SABG assay (n=3). **:*P*<0.01.
